# Exploring the half-metallic ferromagnetism, dynamical and mechanical stability, optoelectronic and thermoelectric properties of K_2_NaMI_6_ (M = Mn, Co, Ni) for spintronic applications

**DOI:** 10.1038/s41598-023-39230-2

**Published:** 2023-08-07

**Authors:** Danish Abdullah, Dinesh C. Gupta

**Affiliations:** Condensed Matter Theory Group, School of Studies in Physics, Jiwaji University, Gwalior, 474011 India

**Keywords:** Materials science, Physics

## Abstract

The structural stability, optoelectronic and magnetic characteristics of K_2_NaMI_6_ (M = Mn, Co, and Ni) halide double perovskites have been demonstrated to be explained using density functional theory computations. The prominent generalized gradient approximation and integration of the mBJ potential are implemented to estimate the exchange–correlation potential, which is the only unidentified parameter in the state-of-the-art formulism. The structural optimization, mechanical stability criteria, and tolerance factor demonstrate the reliability of the double perovskites in a cubic structure with Fm3m symmetry. The elastic constants facilitated mechanical stability and revealed the brittle nature of these double perovskites. The spin-polarized electronic band profile and the behaviour of the dielectric constant and absorption coefficient in the spin-up and down channels show the presence of half-metallic nature in these materials. Additionally, we examined magnetism and the genesis of the half-metallic gap in this article. The half-metallic and magnetic properties are attributed to the unpaired electrons in the split d-orbitals of the M-sited elements in the crystal field. The Mn-, Co-, and Ni-based double perovskites were found to possess total magnetic moments of 4 μB, 4 μB, and 1 μB, respectively, with the transition metal atoms comprising up the majority of this magnetic moment. The Fermi level’s perfect spin polarisation promotes the potential application of double perovskites in spintronic technology.

## Introduction

Materials possessing half-metallic properties with completely spin-polarized conduction electrons and ferromagnetic critical temperatures (Tc)^1^ above ambient temperature are finite in nature. This unique combination of qualities has been demonstrated by two groups of materials: double perovskites and Heusler alloys. As a result, they can make an enormous breakthrough in spintronics by designing spin injectors and tunneling magnetoresistance devices^2,3^. We rely heavily on double perovskites, which have gained great interest due to their close affinity with ternary perovskites. The perovskite family possesses a wide range of amazing features, including high-Tc superconductivity, massive magnetoresistance, and ferroelectricity^4^. Furthermore, A_2_BB′X_6_ double perovskites are a combination of 2 distinct ternary perovskites ABX_3_ and AB′X_3_ organized in a three-dimensional (3D) sequence. The enhanced portability of implementing two different transition metal ions in double perovskites introduces fascinating material investigation opportunities, such as pairing high spin–orbit coupling and strong connection^5^ by merging 5d and 3d transition metals. Moreover, half-metallic ferromagnetic (HMFM) is essential in spintronics since it presents a metallic character in one spin channel and semiconducting or insulating property in another, resulting in 100% spin polarisation at the Fermi level^6,7^. Cubic perovskites dependent on transition elements are fascinating members of the half-metallic ferromagnetic family^8–10^. A recent analysis reported cubic halide double perovskite Cs_2_NaMCl_6_ (M = Mn, Co, Ni)^11^ to be a half-metallic ferromagnet. The possible root of ferromagnetism is due to the hybridization between the I-p and M-d orbitals and the fluctuation in dielectric constant, absorption coefficient, and optical conductivity with energy exhibits photon absorption in the visible–UV region, as well as an appearance of a half-metallic nature. Similarly, Cs_2_NaMCl_6_ (M = Ti, V)^12^ has a good thermoelectric response and has a figure of merit zT that is almost identical to one and is a half-metallic ferromagnet. The halide double perovskite Cs_2_KXCl_6_ (X = Co, Ni) and Rb_2_NaCoF_6_^13,14^ are also half-metallic ferromagnets with a good response to optical characteristics. BaMO_3_ (M = Mg, Ca), KMgO_3_, and REMnO_3_ (RE = Ce, Pr)^15–17^ are new half-metal ferromagnetic materials for spintronic applications.

This study examines the structural, optoelectronic, magnetic, and elastic properties of a cubic K_2_NaMI_6_ (M = Mn, Co, Ni) compound employing realistic density functional theory and the PBE-GGA and mBJ approximation. These compounds exhibit half-metallic ferromagnetism in their cubic phase. This research aims to examine structural stability, photovoltaic and thermoelectric applications, and the genesis of half-metallicity in these materials. Inspire by the above similar discussed materials we explored the cubic phase of the K_2_NaMI_6_ combination because, as far as we were aware, there are no data on its electrical, elastic, optical, magnetic, and transport properties. The high value of the absorption coefficient makes its use for optical implementations and the sophisticated value of the thermoelectric figure of merit zT makes it viable for thermoelectric efficiency which is a necessary component for an eco-friendly environment. These survey results might pave the path for the use of these materials in spintronics.

### Computational

The ground-state energy, electronic, and magnetic properties of the K_2_NaMI_6_ (M = Mn, Co, Ni) perovskites were assessed through spin-polarized calculations employing the Wien2k simulation package. Optimizing the crystal energy concerning volume can be used to numerically predict the stability and ground-state structure. To improve the structure, the Birch–Murnaghan equation^18,19^ of state was utilized

$$E\left( V \right) = E_{0} + \frac{{9B_{0} V_{0} }}{16}\left\{ {\left[ {\left( {\frac{{V_{0} }}{V}} \right)^{\frac{2}{3}} - 1} \right]B_{0}{\prime} + \left[ {\left( {\frac{{V_{0} }}{V}} \right)^{\frac{2}{3}} - 1} \right]^{2} \left[ {6 - 4\left( {\frac{{V_{0} }}{V}} \right)^{\frac{2}{3}} } \right]} \right\}$$

E_0_, B_0_, V_0_, and ${\text{B}}_{0}^{\prime }$ indicate the relaxed state's energy, bulk modulus, volume, and pressure derivatives of B_0_, accordingly. For the Kohn–Sham equation to be solved, both the potential from the core and valence electrons were considered. The exchange–correlation impact was approximated by employing the well-known generalized gradient approach (GGA)^20^. Further, the magneto-electronic property of perovskites was analyzed using the Tran-Blaha-modified Becke-Johnson potential (mBJ)^21^. The unit cell volume is partitioned by muffin-tin spheres (with R_MT_ radii) about the atomic sites and interstitial space. The response of electrons inside the muffin-tin sphere and in interstitial space was addressed by employing a linearized augmented plane wave basis set. The plane wave cut-off in interstitial space was altered to R_MT_K_Max_ = 7, whereas the atomic-like wave functions within spheres have been extended to l_Max_ = 10. The interval between the core and valence states was chosen to be -6.0 Ry (cut-off energy), and the irreducible Brillouin zone was incorporated using a k-mesh of 1000 points. While the charge converged up to 0.0001e and the energy convergence has been 0.0001 Ry.

### Structural stabilities

**Figure 1:**
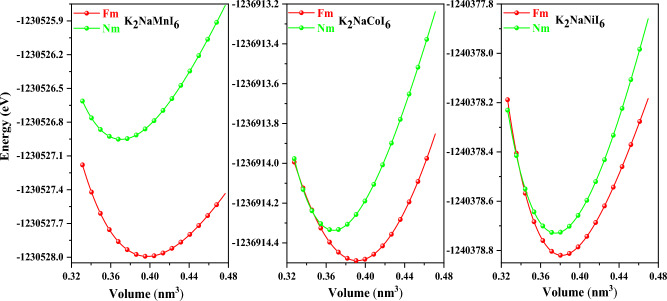
Optimization plots of investigated double perovskites K_2_NaMI_6_ (M = Mn, Co, Ni).

**Figure 2:**
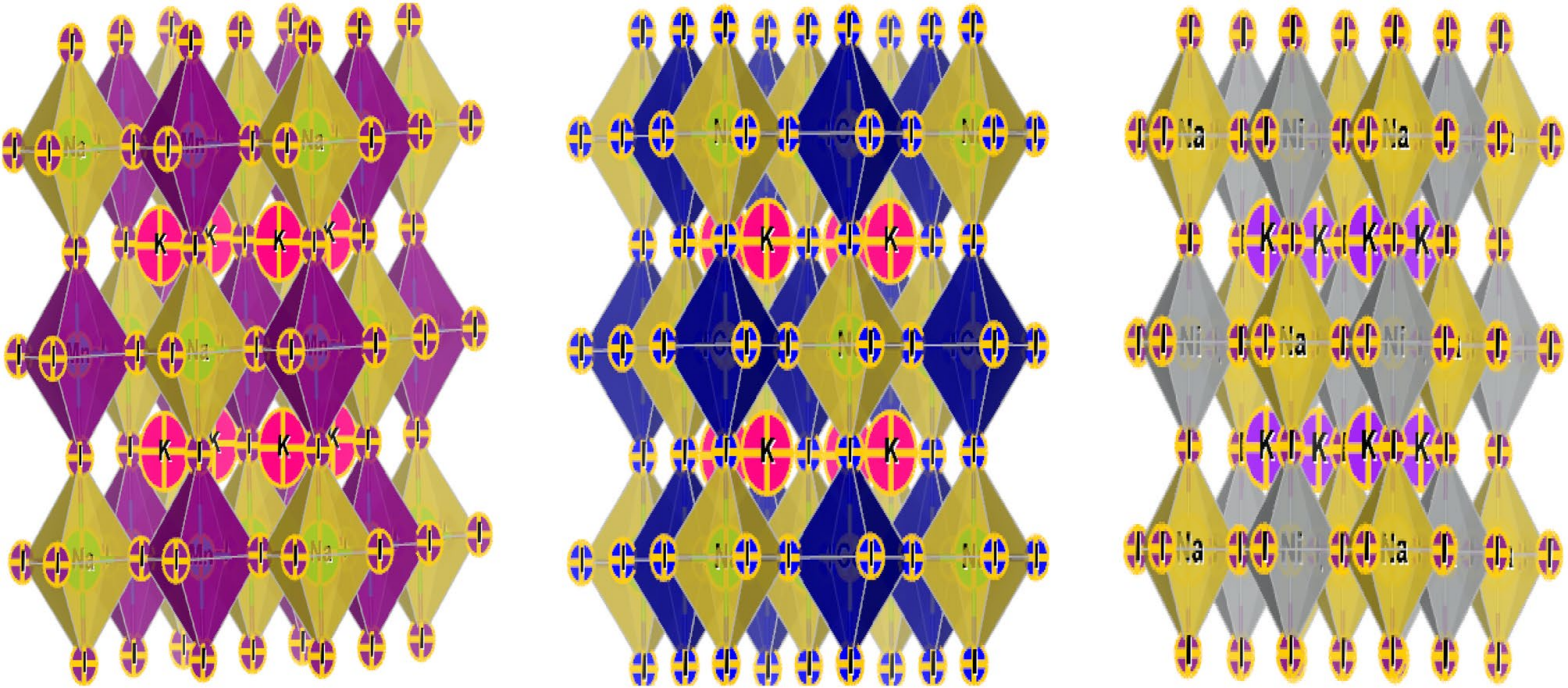
The crystal structure of the compound K_2_NaMI_6_ (M = Mn, Co, Ni).

The double perovskite materials K_2_NaMI_6_ (M = Mn, Co, Ni) with the stoichiometric formula A_2_BB′X_6_ crystallize in a cubic shape with the space group Fm3m (225). The components (atoms) retain their respective position. The bulkier cation possesses 12-fold coordination and dwells in the body-centered cubic system with a Wyckoff position at (0.25, 0.25, 0.25). The enduring B(Na) and B′(M) atoms have fractional coordinates (0.5, 0.5, 0.5), and (0, 0, 0) are positioned in the middle. Furthermore, the B and B′ atoms are settled within the enclosures and are encompassed by the X atoms, which have a geometrical position at the corner locations (0.25, 0, 0), producing varying BX_6_ and B′ X_6_ octahedrons, as depicted in Fig. 2. It is apparent that the Na/M-atoms are surrounded by octahedra of I-atoms, whereas K is coordinated by 12 I-atoms. Moreover, Birch Murnaghan’s equation of state demonstrates the accuracy of the relaxed lattice constants^22,23^. According to the literature survey and visual representation presented in Fig. 1, the minimum ground state energy for the given materials is explicit in the ferromagnetic phase. However, as displayed in Table 1, the stabilization curves for the specified compounds exhibit significant properties like the lattice constant a_0_ (nm), volume in (a.u^3^), bulk modulus (B), a derivative of the bulk modulus (B′), and crystal-free minimum energy (Ry). Moreover, taking into account the formation enthalpy and tolerance factor mentioned in Table 1 is vital when assessing the structural stability of these perovskites^24^. If the estimated result is between 0.9 and 1.0, it means that every atom is precisely aligned at its individual places, leading to an ideal cubic structure. The tolerance factor (τ) is highly dependent on the crystal structure. Because the 12-folded cation is too tiny to occupy its position (τ = 0.71–0.9), an orthorhombic or rhombohedral structure results. If τ is greater than one, the A-site cations become larger to accommodate their sites, leading to hexagonal formations. Different structures are generated for τ = 0.71. The current work demonstrates that K_2_NaMI_6_ (M = Mn, Co, Ni) defines structural stability in the Fm-3 m space group with tolerance factor values in the cubic range (0.9–1). In Mn > Co > Ni-based double perovskites, it is apparent by contrasting the unit cell volume that the lattice constant decreases. This is due to the fact that atomic size decreases. Figure 2 depicts the structure of the unit cell. It is obvious the Na/M atoms are contained within the octahedron of I atoms, with I being coordinated by 12 atoms.

**Table 1:** The Lattice constant a_0_ (nm), Volume (nm^3^), Bulk modulus B (GPa), a derivative of the Bulk modulus B′ (GPa), Energy (eV), Formation of enthalpy (ΔH)(eV), Tolerance factor (τ_F_) and Cohesive energy (Echo) (eV) were evaluated for K_2_NaMI_6_.

Compounds	a (nm)	V (nm^3^)	B (GPa)	B′	E_0_ (eV)(FM)	ΔH	τ_F_	Echo (eV)
K_2_NaMnI_6_	1.167	0.397	16.42	5.10	− 1,230,527.86	− 1.26	0.91	1.64
K_2_NaCoI_6_	1.161	0.391	13.99	4.22	− 1,236,913.20	− 1.28	0.91	1.74
K_2_NaNiI_6_	1.152	0.381	17.09	6.69	− 1,240,378.75	− 1.29	0.91	1.75

The enthalpy of formation is negative on being calculated, which affirms the energetically and thermodynamically stability of the given compound. Therefore, the given compound is stable as revealed in the Table 1.

The energy required to separate a solid into its constituent atoms or significant structural units is known as cohesive energy (Echo). When it comes to compound stability, this energy is vital. The stronger a compound's stability, the higher its cohesive energy. This energy represents how tightly atoms in a material are bound together.

The cohesive energy for double perovskites can be computed as

$$E_{coh}^{{A2BB^{\prime } X6}} = \frac{{2E_{atom}^{A} + E_{atom}^{B} + E_{atom}^{{B^{\prime } }} + 6E_{atom}^{X} - E_{{A2BB^{\prime } X6}} }}{10}.$$

The evaluated value of the given double perovskite K_2_NaMI_6_ is displayed in a Table 1. The results show that the atoms are firmly bound together to form the crystal.

### Phonon stability

**Figure 3:**
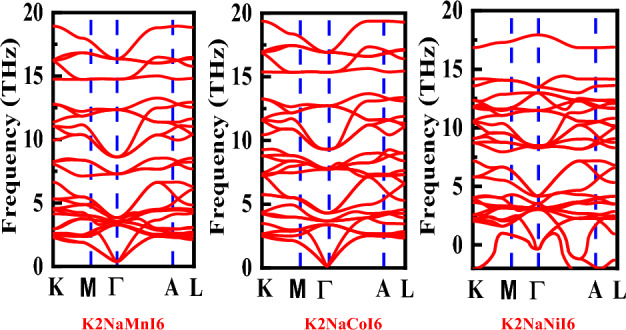
Phonon spectrum calculation for cubic double perovskite K2NaMI6 (M = Mn, Co, Ni).

We executed self-consistent computations on the optimized unit cells to obtain ground state wave functions with a more confined convergence threshold of 10^–18^ for potential residual. The adequate effect of density functional perturbation theory (DFPT) as encoded in Quantum espresso^25^ has been determined in the present scenario to evaluate the dynamical stability within the primitive unit cells of K_2_NaMnI_6_, K_2_NaCoI_6_, and K_2_NaNiI_6_ perovskites and evaluated using mBJ potential and carried out self-consistent perturbation computations with the convergence threshold of 10^–18^ for the potential residual to obtain the dynamic matrix. For the phase stability of K_2_NaMI_6_ scenarios, the quantified phonon dispersion curves along the high-symmetry directions in the Brillouin zone appear in Fig. 3. The stability of the compound K_2_NaMI_6_ (M = Mn, Co, Ni) has been evaluated using a phonon spectrum computation. In addition, a proper understanding of the lattice-dynamical characteristics of solid materials—where phonons play an intriguing role—is necessary to comprehend many aspects of materials. The phonon dispersions curves were obtained for that purpose once lattice dynamics computations were completed. In theory, a crystal lattice with n atoms per unit cell will have 3 n branches, 3 of which will be acoustic, while the remaining n will be optical. According to the criterion for a structure's dynamical stability, all frequent vibration modes must exhibit actual and finite frequency. Our findings are displayed in Fig. 3. Imaginary frequencies are absent in K_2_NaMnI_6_ and K_2_NaCoI_6_ based perovskites, indicating their dynamical stability. Additionally, it is evident that the K_2_NaNiI_6_ structure has imaginary frequencies^26^, which are below zero in the ordinate scale of Fig. 3. As a result, based on the current computations, K_2_NaNiI_6_ is dynamically unstable in this structure because it shows a small negative frequency.

### Elastic properties

The computed elastic constants are vital for comprehending the material's structural reliability and certain other mechanical properties. It expresses the material's behavior when pressure is applied to the material. The cubic structure has 3 (three) elastic constants C_11_, C_12_, and C_14_ that can depict the material's mechanical properties. Table 2 shows the simulated elastic constants of lead-free double perovskites K_2_NaMI_6_ (M = Mn, Co, Ni). To ensure the validity and reliability of our computation, we incorporate Born-Huang stability rules^27^ for cubic crystals: C_11_ > 0; C_44_ > 0; C_11_ + 2C_12_ > 0; C_11_ − C_12_ > 0. The observation of our calculations suggests that these titled double perovskites K_2_NaMI_6_ (M = Mn, Co, Ni) meet the Born–Huang stability criteria, confirming these perovskite materials are stable. The shear modulus (G)^28^ of a material, which offers information about its rigidity, is used to evaluate its resistance to reversible deformation under shear stress. As a result, G is computed using two models: Voigt (G_V_)^29,30^ and Reuss (G_R_)

$${\text{G}}_{{\text{V}}} = \frac{1 }{5}\left( {3{\text{C}}_{44} + {\text{C}}_{11} - {\text{C}}_{12} } \right)$$

$${\text{G}}_{{\text{R}}} = 5\left( {{\text{C}}_{11} - {\text{C}}_{12} } \right){\text{C}}_{44} /4{\text{C}}_{44} + 3\left( {{\text{C}}_{11} - {\text{C}}_{12} } \right).$$

**Table 2:** Elastic constants (C_11_, C_12,_ and C_44_ in GPa), Bulk modulus (B in GPa) Voigt’s Shear (G_V_ in GPa), Reuss’s Shear (G_R_ in GPa) modulus, Shear modulus (G in GPa), Young’s modulus (Y in GPa), Micro hardness (H) and Cauchy’s pressure (C_P_) in GPa, Anisotropy ratio (A), Pugh’s ratio (*B*_*0*_*/G*) and Poisson’s ratio (ν).

Parameter	K_2_NaMnI_6_	K_2_NaCoI_6_	K_2_NaNiI_6_
C_11_	52.22	42.98	39.90
C_12_	21.31	19.34	18.62
C_44_	20.43	18.62	17.96
B = B_V_ = B_R_	31.62	27.22	25.71
G_V_	18.09	15.14	14.08
G_R_	18.44	15.9	15.02
G	18.26	15.51	14.56
Y	45.96	39.12	36.74
H = 0.92 (B/G)^1.3137^ G^0.708^	21.80	19.96	19.32
Cp	0.88	0.72	0.66
A = 4C_44_/(C_11_–C_12_)	1.32	1.57	1.69
B_o_/G	1.72	1.74	1.74
ѵ	0.25	0.25	0.25

And, Hill ^31^ (G_H_) computed the arithmetic mean of G_V_ and G_R_:

$${\text{G}}_{{\text{H}}} = {\text{G}}_{{\text{V}}} + {\text{G}}_{{\text{R}}} /2.$$

The measured G_H_ values depicts that K_2_NaMnI_6_ has the maximum shear modulus (18.26 GPa), implying that it is harder than the other materials. Young's modulus (Y) can be used to evaluate a material's reaction to linear deformation^32^, which implies that a material's toughness is inversely correlated with the value of Y. Owing to the connection, the tougher the material is, the higher the value of Y.

$${\text{Y}} = 9{\text{BG}}_{{\text{V}}} /3{\text{B}} + {\text{G}}_{{\text{V}}}$$

The greater Y value (45.96 GPa) for K_2_NaMnI_6_ signifies that it is the toughest of these compounds. A material's ductile or brittle character can be distinguished using Pugh's ratio (B/G)^33^; a specimen is ductile if the ratio is more than 1.75; otherwise, it is brittle. Our analysis of the data shows that these materials are brittle because their B/G values are below the critical value of 1.75. Cauchy pressure can be used to define a material's capacity for bonding as well as its brittle or ductile nature.

The following equation explores the value of Cauchy pressure.

$${\text{C}}_{{\text{P}}} = {\text{C}}_{12} - {\text{C}}_{44}$$

Higher Cauchy pressure affects ionic bonding, while lower values reveal directional bonding (covalent bonding). These compounds are brittle by nature, as shown by the C_P_ values in Table 2. Compressibility is assessed using Poisson’s ratio v, which gauges the contribution of neighbouring longitudinal strain to non-axial tensile stress:

Exploring Poisson’s ratio can also acknowledge the elastic properties of a composite material. The difference between ductile and brittle characters is denoted by the index value of 0.26. Double perovskites exhibit a brittle character below their index value while being ductile above it.

### Electronic band structure

**Figure 4:**
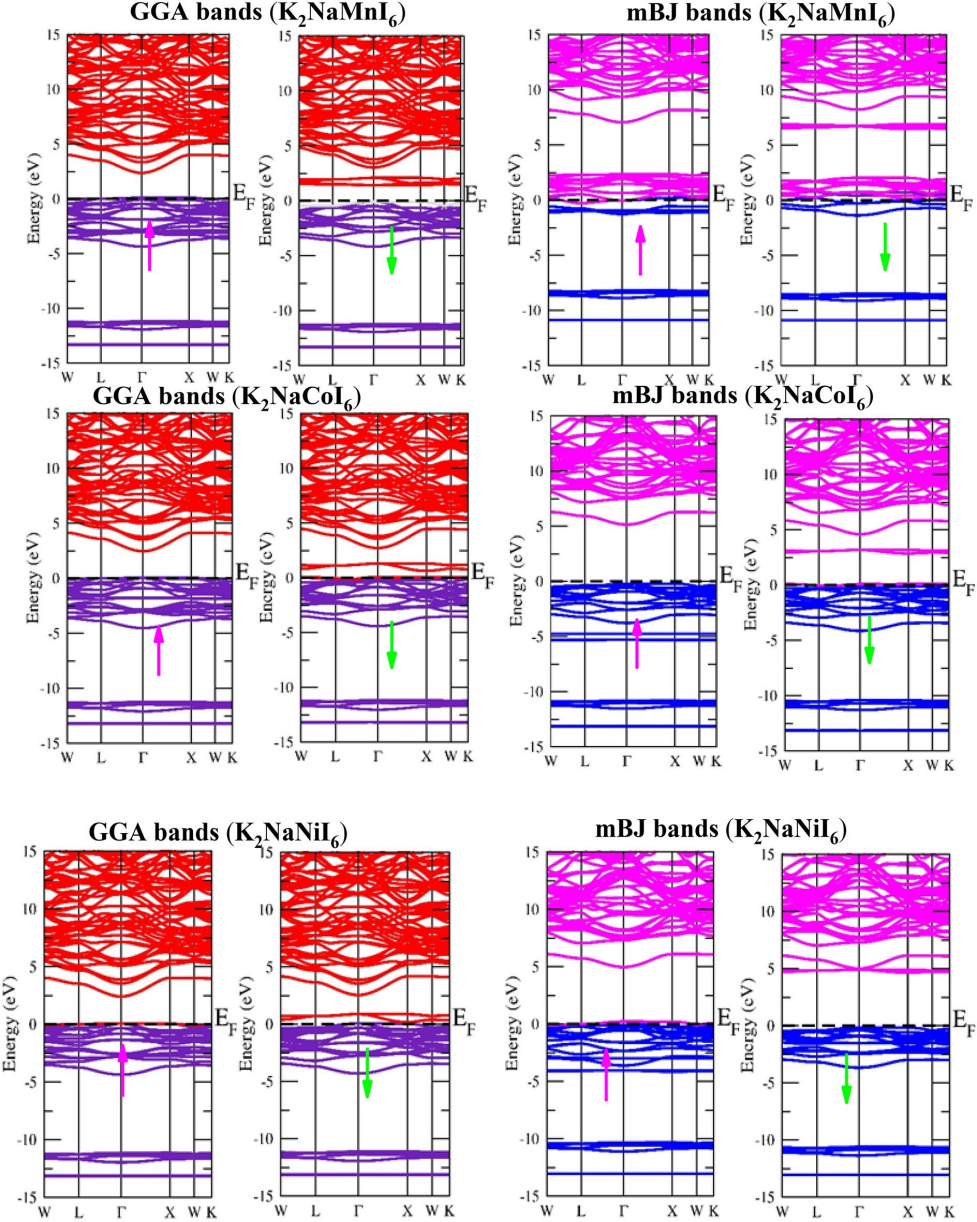
Calculated Band structures utilizing GGA and mBJ approximations of K_2_NaMI_6_ (M = Mn, Co, Ni).

A material’s electronic and magnetic properties can be analyzed to determine its feasibility for technological applications. The electronic band structure (BS) operates a vital role in describing the nature of a material whether a material is a conductor, semiconductor, or insulator. If the electronic states between the valence band (VB) and conduction band (CB) overlap at the fermi level this results in metallic behavior or if there exists a forbidden gap between VB and CB this results in a semiconductor or insulator. The forbidden gap between VB and CB can be reduced by applying the temperature that is band gap and temperature are inversely proportional to each other. For the mentioned double perovskites, we address the electronic band structure, total density of states, and partial density of states (TDOS and PDOS). Spin-polarized calculations are used to interpret the electrical behavior of up and down spin channels. Figure 4 depicts the band structure as evaluated using the spin-polarized GGA and mBJ techniques. From the BS, it is apparent that the spin-up states of Mn and Ni compounds and the spin-down of Co detect metallic properties since the energy states in each channel surpass the Fermi level, which is set at 0 eV. There are no energy states at the fermi level when in the spin-down channel except in the case of Cobalt which manifests the metallic aspect in the spin-down channel. Furthermore, for Mn (Ni) based materials, the prohibited band gap between the conduction band minima and the valance band maxima is 4.41 (4.87 eV) indicating that the spin-down channel is semiconducting in nature, and for cobalt-based material semiconducting gap (5.3 eV) exists in the up channel. The occurrence of transition element d-valance electrons in K_2_NaMI_6_ may render the GGA inefficient for the compounds which in turn makes the energy band gap underrated. As a result, modified Becke Johnson potentials are combined in GGA to more precisely describe band structure.

**Figure 5:**
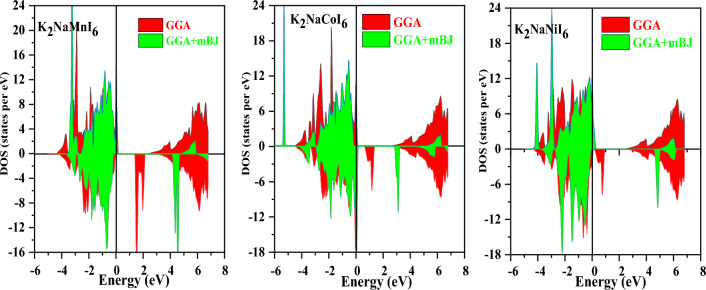
Calculated results of TDOS for K_2_NaMI_6_ (M = Mn, Co, Ni) using the GGA and mBJ approximations.

The electronic properties of the K_2_NaMI_6_ (M = Mn, Co, Ni) double perovskites are modified by both GGA and GGA + mBJ approximations and are illustrated by the total density of states (TDOS) as shown in Fig. 5. The band gap in the spin-down semiconducting channel (Mn, Ni) and spin-up channel of Co has broadened as a response to the electronic states of K_2_NaMI_6_ drifting farther from the Fermi level.

**Figure 6:**
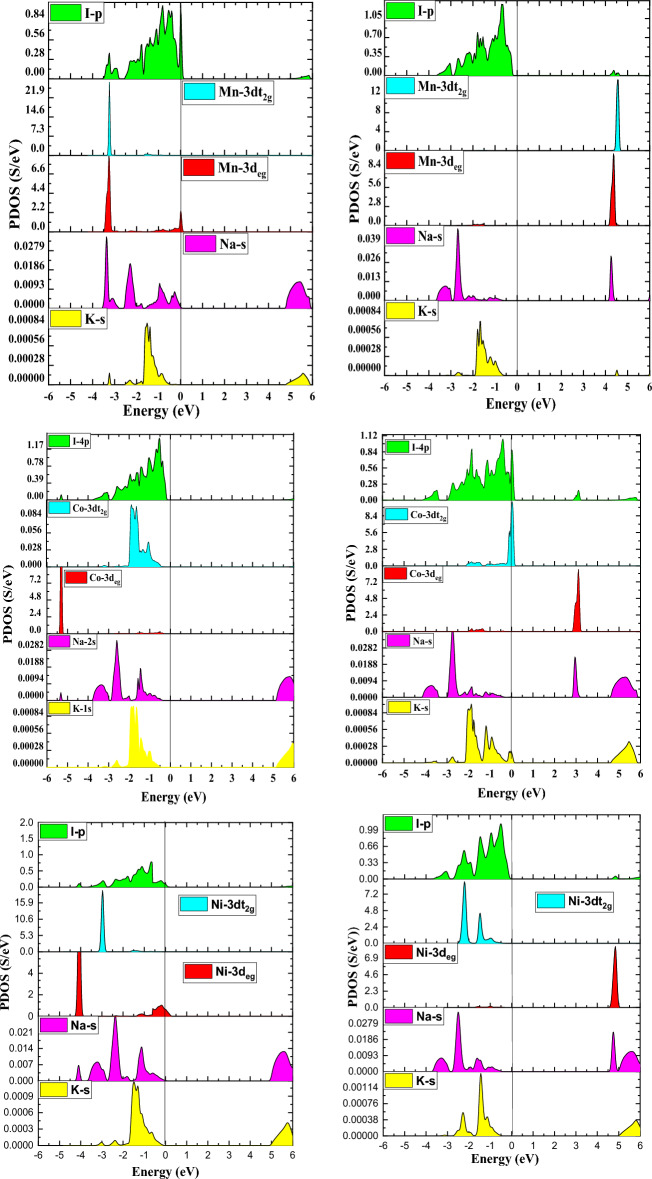
Calculated partial density of states using mBJ approximation for material K_2_NaMI_6_ (M = Mn, Co, Ni).

Furthermore, we assessed the partial density of states (PDOS) to exclusively examine the band structure and framed the energy states affiliated with the valence and conduction bands. As demonstrated in Fig. 6, the I-atom promotes the greatest layout of the valance band. The d-states of the transition atom are vital and have a profound impact on electronic performance. Assessing the PDOS of the three double perovskites discloses that the transition atoms’ d-states are orientated diversely across the Fermi level, which contributed to their half-metallic nature. In the spin-up channel’s valance band of Mn, p–d hybridization emerges between the dt_2g_ states of Mn and I. The Mn-deg states at the Fermi level are accountable for the metallic nature of the Mn-based double perovskite. In addition, all of the d-states of Mn in the spin-down channel are shifted from the Fermi level form the conduction band minima. In the Co-based double perovskite Both the dt_2g_ and de_g_ states of the spin-up channel lie below the Fermi level and are involved in p-d hybridization. While the dt_2g_ states are at the Fermi level in the spin-down channel reflects metallic character whereas the de_g_ states are unoccupied lie above the Fermi level reveals the semiconducting nature, leading to the half metallicity of the given compound. The dt_2g_ states, on the other hand, are totally filled for both spin channels in the Ni-based double perovskite and dwell in the valance band. The de_g_ states in the spin-up channel at the Fermi level contribute to the metallic character, while the conduction band minima appear in the spin-down state. However, the dt_2g_ states are entirely filled for both spin channels and are positioned in the valance band in Ni-based double perovskite. The deg states produce metallic character in the spin-up channel at the Fermi level, whereas in the spin-down state, they support the conduction band minima. The total magnetic moment for the Mn-, Co-, and Ni-based double perovskites is 4μB, 4μB, and 1μB, respectively. The prevalence of half-metallicity is also revealed by the integral magnetic moment.

### Source of half metallic character

The M-site atoms appear in the I-atoms’ octahedral environment, as specified in the structural details. The degeneracy of the Mn/Co/Ni d(d_xy_, d_yz_, d_zx_, d_x_^2^_–y_^2^, d_z_^2^) orbitals, which have been separated into dt_2g_ (d_xy_, d_yz_, d_zx_) and d_eg_ (d_x_^2^_–y_^2^, d_z_^2^) sets, is diminished by the crystal field caused by M-I Coulomb interactions. According to Hund's rules, the d_eg_ state can only hold an aggregate of 4 electrons (2↑ + 2↓), whereas the dt_2g_ state has an intake ability to hold 6 electrons (3↑ + 3↓). The electrons have a choice of concerning forming a low-spin state, in which d_eg_ states are filled only after the dt_2g_ states are completely occupied, or a high-spin state, in which higher energy de_g_ levels are filled before pairing appears in the dt_2g_ orbitals. The crystal field splitting energy (CFS) and pairing energies investigate whether a high-spin state or a low-spin state will occur. The high-spin state can emerge only when the splitting energy is less than the pairing energy. The CFS is impacted by the size of the central atom and the number of electrons. The splitting is impacted by the ligand type; strong field ligands may cause extensive splitting. The creation of the high-spin state relies on the oxidation number, electron number, and size of the transition anion when weak field ligands like Cl/Br/I appear because the CFS is small. The expected oxidation states in these double perovskites are K_2_^1+^Na^1+^M^3+^I_6_^1-^. Mn^3+^, Co^3+^, and Ni^3+^ have 4, 6, and 7 valance electrons, respectively, in their d states. The Mn^3+^ d state has an electron occupancy of 3t_2g_ (↑), 1eg (↑), 0t_2g_ (↓), and 0e_g_ (↓), leading to a high-spin state with S = 2. The conduction band minima are generated by the vacant d states in the spin-down state. As shown in Fig. 6 the deg state in the spin-up channel is partially filled and resides at the Fermi level. The Co-based double perovskite’s DOS plots make it remarkably clear that all of the d states in the spin-up channel are inhabited. The Fermi level is attained by the dt_2g_ states in the spin-down channel. As a result, the electrons inhabiting the valance states of the Co^3+^ states are 3t_2g_ (↑), 2e_g_ (↑), 1t_2g_ (↓), and 0e_g_ (↓), resulting in four electrons unpaired with S = 2. The occupancy suggests that the d-orbitals in the spin-up channel are filled, producing the valance band. While partially filled dt_2g_ states in the anti-parallel spin channel contribute the associated spin channel a metallic aspect. The number of electrons at the center atom grows as one moves from Mn to Ni, while the size diminishes. Ni3^+^ has the electrical configuration 3t_2g_ (↑), 3t_2g_ (↓), 1e_g_ (↑), and 0e_g_ (↓), which left one unpaired electron and so S = 1/2. When Ni is the most central atom, the spin state turns from high to low. Each unpaired electron possesses a magnetic moment approaching 1 μB, therefore the total magnetic moment for the Mn-, Co-, and Ni-based double perovskites is 4 μB, 4 μB, and 1 μB respectively.

### Thermodynamics

We have integrated a quasi-harmonic approximation of the Debye model (QHM)^34^ to examine the thermodynamic reliability of these perovskites K_2_NaMI_6_ (M = Mn, Co, Ni). The QHM massively represents the stability of these different quantities, encompassing the specific heat at a constant volume (C_v_), Debye temperature, and Gruneisen parameter (γ), in the temperature and pressure range of 0–550 K and 0–5 GPa, this type of model is desirable and acknowledged as being efficient in predicting the findings in a certain temperature and pressure ranges.

**Figure 7:**
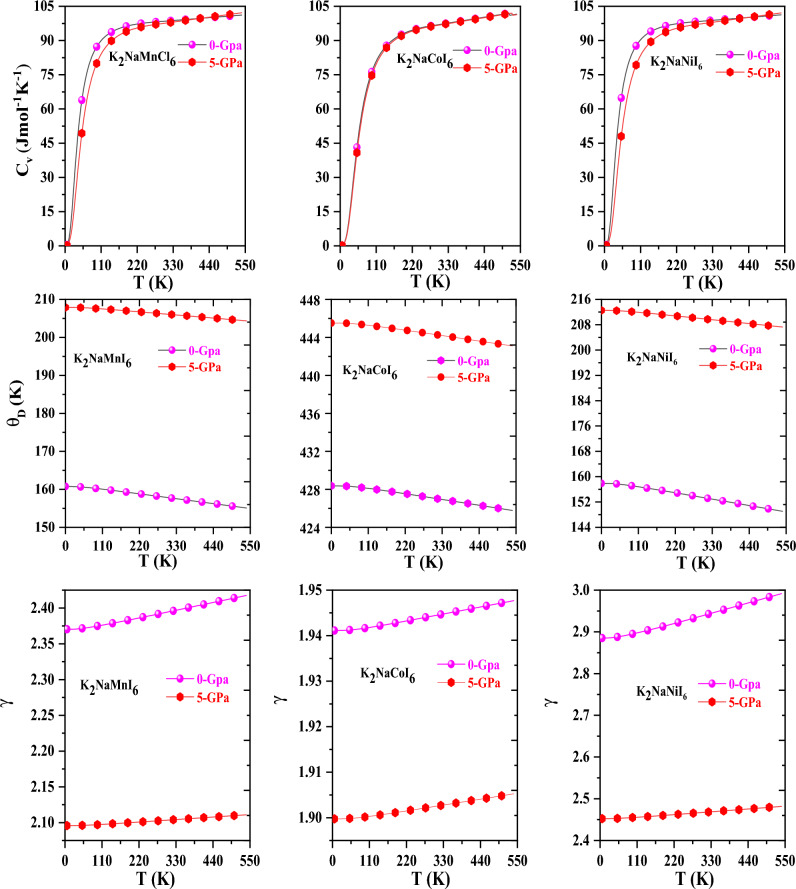
Variation of specific heat (Cv), Debye temperature (θ_D_), and Gruneisen parameter (γ) with temperature.

We proceeded by demonstrating the specific heat of each material at constant volume (C_V_), which belongs to the critical aspects that affect the material’s behavior. The current materials have a flatter trajectory for heat transmission at room temperature due to the T^3^ relation. At elevated temperatures, the graphical deviation of the specific performance of the entitled compound has a drift toward the Dulong petit limit, as depicted in Fig. 7. The high-temperature constraint indicates that the change follows Delong-law Pettit’s^35^.

Among solids' most essential thermodynamic qualities is the Debye temperature (θ_D_)^36^. It is the maximum temperature at which the particles (constituents) produce correlated vibrations and the vibrations attain their highest achievable frequencies. It is an effective indicator of material hardness; materials with a significant θ_D_ have high stiffness and are relatively very robust than materials with a low θ_D_. Figure 7 depicts θ_D_ as a function of temperature, it is recognizable that θ_D_ diminishes as temperature increases. Thermal expansion and anharmonicity are modest at low temperatures, resulting in a virtually constant Debye temperature. At low temperatures, the high-frequency modes can be thought of as locked; only the sound waves are activated. As a result of the θ_D_ decreasing with temperature, the atoms' vibrational frequencies change as a function of temperature. K_2_NaMNI_6_, K_2_NaCoI_6_, and K_2_NaNiI_6_ have Debye temperatures of 157.87 (206.11)K and 427.09 (444.34)K and 153.44 (209.90) K at 300 K and 0 (5) GPa respectively, at ambient temperature. The perovskites can be operating for applications at higher temperatures, depending on the high value of the Debye temperature.

Moreover, as depicted in Fig. 7, we have simulated the Gruneisen parameter (γ), which describes the anharmonicity and presents a clear explanation of the phonon frequency modes. At lower temperatures, the Gruneisen parameter (γ) has a gradual ascending exponential pattern; but, at higher temperatures, remains almost constant. However, pressure’s consequence on (γ) has a limited impact on it. The Gruneisen parameter has been assessed to have values of 2.39, 1.94, and 2.93 at 300 K and 0 GPa, respectively.

### Thermoelectric

Due to the expansion of the global economy in recent years, there have been severe energy crises and significant environmental degradation as a result of the massive increase in the utilization of fossil fuels. To determine how effectively thermal energy can be converted into electrical power, the thermoelectric (TE) characteristics of K_2_NaMI_6_ double perovskites are examined. Utilizing TE compounds, excess heat can be turned into electricity to solve the energy shortage and minimize harmful emissions^36–40^. In the 0–1000 K temperature range, we assessed TE parameters such as electrical conductivity (σ), Seebeck (S), thermal conductivity (κ), and figure of merit zT using the BoltzTraP2 code.

**Figure 8:**
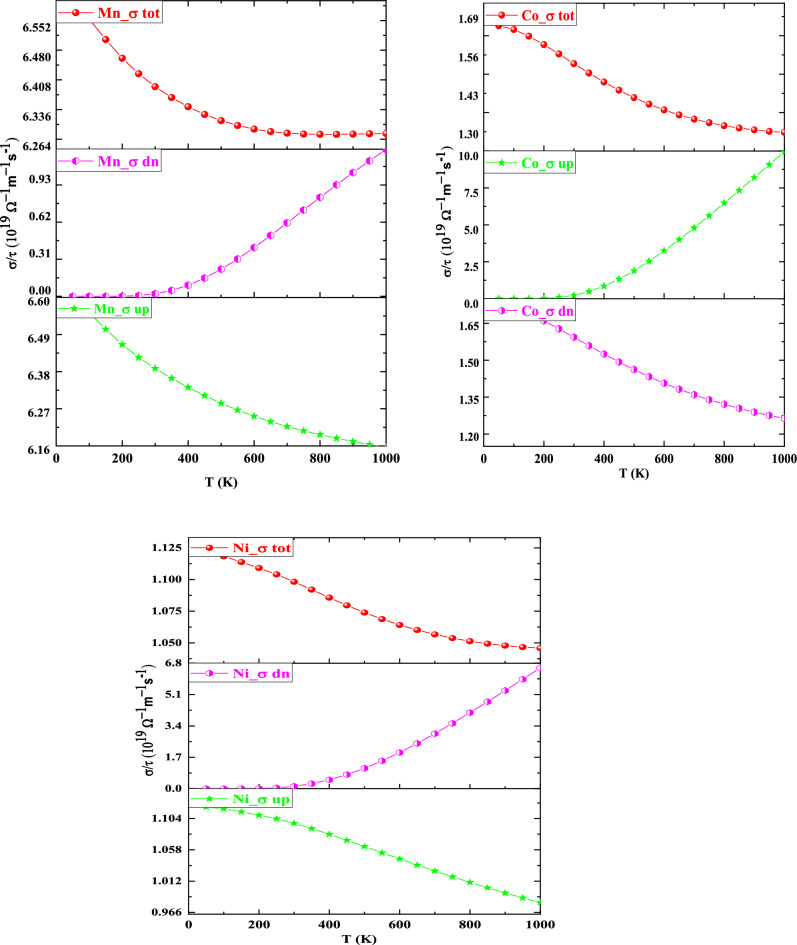
Variation of electrical conductivity (σ) with Temperature for K_2_NaMI_6_ (M = Mn, Co, Ni).

The number of electrons applicable for conduction dictates electrical conductivity (σ). In the Spin-up channel of K_2_NaMnI_6_, K_2_NaNiI_6,_ and the spin-down channel of K_2_NaCoI_6_ the electrical conductivity diminishes with a rise in temperature revealing the metallic behavior in the respective channels as indicated in Fig. 8. At 50 K (1000 K) the simulated electrical conductivity values for Mn, Co, and Ni are 6.62 (6.15) Ω^−1^ m^−1^ s^-1^, 1.72 (1.26) Ω^−1^ m^−1^ s^−1^, and 1.12 (0.98) Ω^−1^ m^−1^ s^−1^. Moreover, in the spin-down of K_2_NaMnI_6_, K_2_NaNiI_6_, and the spin-up of K_2_NaCoI_6_ the electrical conductivity increases with an increase in temperature determining the semiconducting behavior in their respective channels. The calculated values for Mn, Co, and Ni at 50 K (1000 K) are 0.0012 (1.23) Ω^−1^ m^−1^ s^-1^, 0.0017 (9.95) Ω^−1^ m^−1^ s^−1^, and 0.0102 (6.54) Ω^−1^ m^−1^ s^−1^. At 0 K the semiconductor behaves almost like an insulator, however, with rising temperature, the amount of charge carriers increases as they acquire sufficient energy to move from the VB to CB. These compounds have maximum electrical conductivity owing to their exceptionally low resistance, implying that they could be employed to fabricate thermoelectric devices.

**Figure 9:**
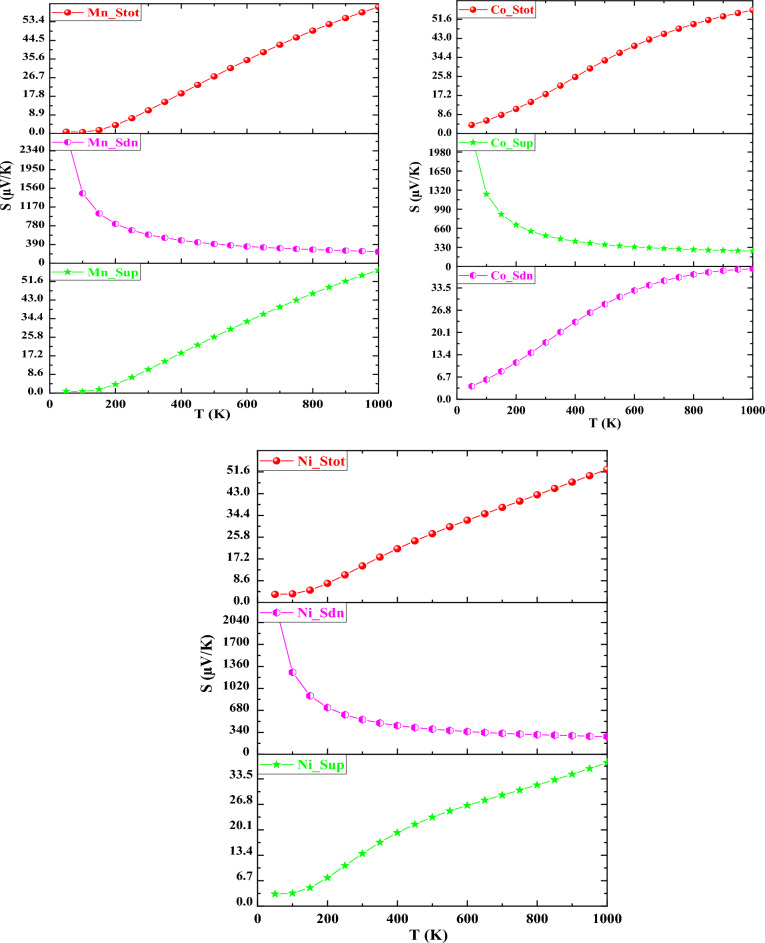
Alteration of Seebeck coefficient with temperature for K_2_NaMI_6_ (M = Mn, Co, Ni).

The Seebeck coefficient (S) explains a material's capability of producing electrical potential. It is a crucial factor for determining the thermoelectric efficiency of a material. The Seebeck coefficient indicates the quantity of voltage generated by a material as a result of a temperature variation across it. Figure 9 shows the Seebeck coefficients (S) of the given halides K_2_NaMI_6_ (M = Mn, Co, Ni). The halide perovskite (Mn, Ni) performs metallically in the case of spin-up, which implies that the Seebeck coefficient rises with an increase in temperature, and for Co, it shows metallic behavior in the down channel thus for the metallic case, the Seebeck coefficient in K_2_NaMnI_6_, K_2_NaNiI_6_, and K_2_NaCoI_6_ grows abruptly from a lowest value of 0.86 (57 μV/K), 3.91 (39.28 μV/K) and 3.20 (37.79 μV/K) along the specified 50 (1000 K) temperature. The exact diminishing nature is experienced in the spin-down scenarios for the two specific perovskites K_2_NaMnI_6_ and K_2_NaNiI_6_ from the lowest temperature of 50 K with a value of 2698.27 μV/K and 2334.19 μV/K towards the known value of 244.19 μV/K and 274.28 μV/K at 1000 K and in case of Cobalt, it shows the decreasing trend in spin up channel depicts semiconducting nature in the respective channel. The calculated Seebeck coefficient value of K_2_NaCoI_6_ at 50 K (1000 K) is 2270 μV/K (265 μV/K). As a result, the interpretation retrieved from the proper Seebeck coefficient ratio values desires to broaden the approach toward thermoelectric applications.

Thermal conductivity (к) is a flow of energy (heat) in response to a temperature difference. The significance of к is comprised of two components. The prime is induced by electronic transport, which is addressed as the electronic part of thermal conductivity (к_e_), and the second is impacted by lattice vibrations (phonons), which is examined as the lattice component of thermal conductivity (к_l_).

$$\upkappa =\upkappa _{e} +\upkappa _{l}$$

**Figure 10:**
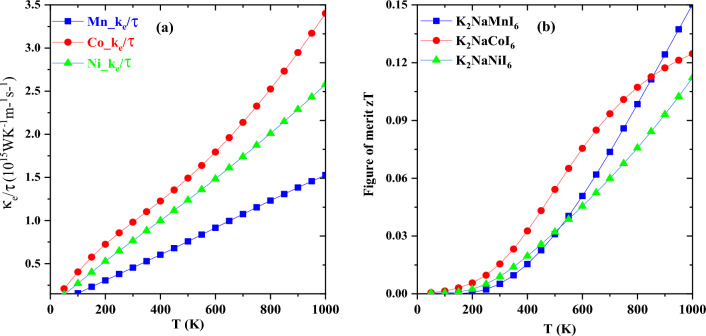
Alteration of electronic thermal conductivity (**a**) and figure of merit zT (**b**) with temperature for K_2_NaMI_6_ (M = Mn, Co, Ni).

At high temperatures, the merit of к_l_ can be discarded since it exhibits an inverse relationship with absolute temperature^41,42^. As a response, we computed the thermal conductivity (electronic component) per unit relaxation time (к_*e*_/τ) for K_2_NaMI_6_ (M = Mn, Co, Ni) as depicted in Fig. 10a. The variation of к_e_/τ concerning temperature reveals the same increasing pattern as that of σ/τ. The Wiedemann–Franz law^43^ explains the linear relationship between electrical and thermal conductivity, which compensates for this behavior. The highest values of к_*e*_/τ were 1.53 × 10^15^ W m^−1^ k^−1^, 3.40 × 10^15^ W m^−1^ k^−1^, and 2.58 × 10^15^ W m^−1^ k^−1^ for K_2_NaMnI_6_, K_2_NaCoI_6_, and K_2_NaNi_6_ respectively.

The figure of merit (zT) is of great importance in scrutinizing the effectiveness of thermoelectric (TE) characteristics. The zT is a thermal efficiency parameter that could be computed utilizing the mentioned calculation^44–46^.

$${\text{zT}} = {\text{S}}^{2}\upsigma {\text{T}}/\upkappa$$

The improved value of S with σ and the low value of κ strengthened the value of zT. At 1000 K, the zT values of K_2_NaMnI_6_, K_2_NaCoI_6_, and K_2_NaNiI_6_ are 0.15, 0.12, and 0.11 respectively (see Fig. 10b). These findings indicate the feasibility of these materials for thermoelectrical applications.

### Optical properties

To ascertain whether material is adequate for an optoelectronic application, it is essential to scrutinize a variety of optical characteristics, including the real (ε_1_(ω)) and imaginary (ε_2_(ω)) portions of the dielectric function, the absorption coefficient α(ω), the optical conductivity σ(ω), and several others.

The dielectric functions used to scrutinize polarization and absorption of electromagnetic radiation within the specific material are numerically conveyed by the relationship ε_1_(ω) = ε_1_ω + iε_2_(ω)^47,48^. In this relationship, the first term stands for the real part ε1ω, which illustrates the polarisation of light while the second term (ε_2_(ω)) performs the imaginary portion of the dielectric function, or term ε_2_(ω), which measures the degree of light absorption. For optoelectronic devices, the strength of the transitions between and within bands determines the absorption and emission of light strengths.

**Figure 11:**
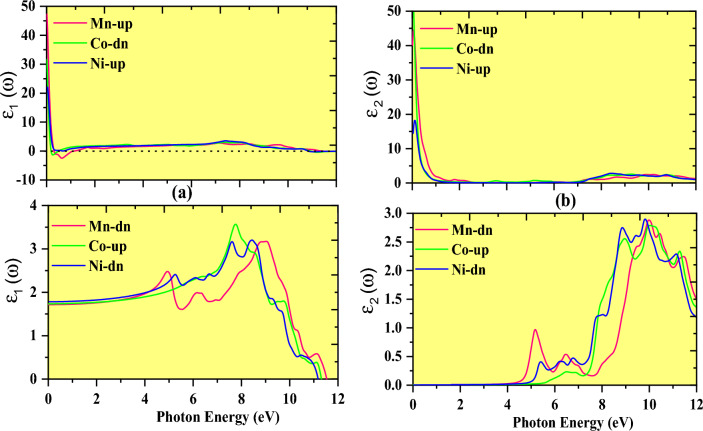
Fluctuation in dielectric constant with photon energy. (**a**) Real part and (**b**) imaginary part of compound K_2_NaMI_6_ (M = Mn, Co, Ni).

The fluctuation of ε_1_(ω) and ε_2_(ω) with reference to photon energy is illustrated in Fig. 11a,b where it is practicable that in the spin channels of Mn-up, Co-down, and Ni-up, the high value shifts negative between 0–1.3 eV for the Mn- and Co-based double perovskites. Incoming electromagnetic energy (photon) is effectively dampened by the double perovskites in the negative range of ε_1_(ω), confirming their flawless metallic character. As shown in Fig. 11a the ε_1_(ω) affects the linear behavior of polarisation and photon energy distribution while traveling across materials in spin channels of Mn-down, Co-Up, and Ni-down, its values rise with increasing photon energy and peak at the resonance frequency. The largest peaks for Mn (Co) and Ni were detected at 9 eV (8.5 eV) and 7.8 eV. As a result, at the resonance frequency, the light is entirely polarised or dispersed. When the frequency of an incoming photon fluctuates significantly from resonance values, the polarisation abruptly lowers and light energy begins to absorb, as represented by the imaginary ε_2_(ω) in the following section.

A key indicator of the functionality of optoelectronic devices is the imaginary part of the dielectric constant, ε_2_(ω), which depicts the photons that perovskites can retain. The optical edge noticed at 0 (zero) eV for Mn-up, Co-down, and Ni-up denotes the metallic character in the admissible spin channel. The Mn-down, Co-up, and Ni-down spin channels could be semiconducting because there aren't any energy levels at the Fermi level, contrary to the existence of peaks other than zero energy. Moreover, Fig. 11b illustrates the factor ε_2_(ω) that controls how much photon energy changes as it passes through a material medium. Interestingly, the critical ε_2_(ω) value plays a crucial role in revealing the threshold energy that characterizes the difference between transmission and absorption. For K_2_NaMI_6_ (M = Mn, Co, Ni), the expected optical and electronic band gaps are in remarkable conformity. The absorption bands' energy values fall within the visible and ultraviolet energy ranges. The existence of an absorption area in the visible range has tremendous potential for the development of solar cells and optoelectronics.

**Figure 12:**
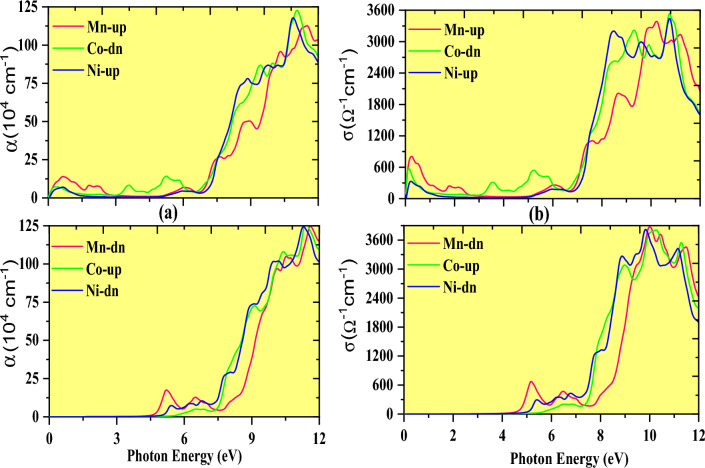
Fluctuation in absorption coefficient (**a**) with photon energy and (**b**) optical conductivity with a photon energy of K_2_NaMI_6_ (M = Mn, Co, Ni).

Figure 12a shows the calculated α(ω) spectrum as a function of energy, indicating how abundant light energy is absorbed by a substance^49^. The energy peaks in the spin-up of Mn and Ni and the spin-down channel of cobalt support metallic character, and the absence of absorption in the energy range below 2.5 eV, suggests these materials are transparent, supporting the semiconducting nature. The absorption coefficient steadily increases in visible and ultraviolet energy ranges, which is greatly affected by inter-band transitions from the VB to CB. These materials are more convenient for solar cell applications due to their immense optical absorption coefficient α(ω) in visible and ultraviolet ranges. However, we saw significant peaks develop in an energy range of 9–11 eV; following the energy value, absorption gradually diminishes, as shown in the absorption plot (Fig. 12a).

The expansion of electrical transmission^50^ to high spectral frequencies is defined as optical conductivity σ(ω). The computed conductivities are shown in Fig. 12b, this material feature is important for optoelectronic devices. Mn-up, Ni-up, and Co-down materials exhibit optical conductivity at lower photon energies, indicating that light absorption in low-energy regions sustains the metallic character in their respective channels. Furthermore, the optical conductivity grows quickly with the increase in photon energy at around 4 eV and reaches the maximal value for the given material at around 10 eV for the down channels of Mn and Ni and in the spin-up of Co. The investigated halides are a viable candidate for implementation in solar cells and other low-energy optoelectronic devices owing to their optical conductivity at this energy level.

## Conclusion

In summary, the simulated structure demonstrates that the cubic phase of the halide perovskite K_2_NaMI_6_ (M = Mn, Co, Ni) is stable. The negative value of the formation energy E_F_ and the positive value of the cohesive energy (E_c_) revealed that the present compound is thermodynamically and energetically stable. The band structure has been specifically acquired using mBJ approximation. From spin-polarized band structure and DOS estimations, it is obvious that all of these compounds are ferromagnetic half metals due to the reasonable band gap in the spin-down channels (Mn and Ni) and up channels of Co, as well as 100% spin polarisation at Fermi level. For K_2_NaMI_6_ (M = Mn, Co, Ni) the absolute spin magnetic moment value is 4 μB, 4 μB, and 1 μB and these magnetic moment values are merely due to the M atoms via strong p-d hybrid. The brittle nature of these compositions is revealed in the computation of Poisson’s ratio (< 0.26) and Pugh’s ratio (1.75) ratio. Diverse optical properties reveal that these materials can be widely used for optical devices/applications owing to their strong absorption and solar cell applications due to their outstanding optical conductivity. By examining the transport properties, advanced thermal efficiency values for K_2_NaMI_6_ are achieved. These findings demonstrate that K_2_NaMI_6_ can be employed effectively for thermoelectric devices as well as spintronics applications.

## Data Availability

The dataset created and/or analyzed during the current study is available upon reasonable request from the corresponding author.
